# *TLR9* -1486T/C and 2848C/T SNPs Are Associated with Human Cytomegalovirus Infection in Infants

**DOI:** 10.1371/journal.pone.0154100

**Published:** 2016-04-22

**Authors:** Edyta Paradowska, Agnieszka Jabłońska, Mirosława Studzińska, Katarzyna Skowrońska, Patrycja Suski, Małgorzata Wiśniewska-Ligier, Teresa Woźniakowska-Gęsicka, Dorota Nowakowska, Zuzanna Gaj, Jan Wilczyński, Zbigniew J. Leśnikowski

**Affiliations:** 1 Laboratory of Molecular Virology and Biological Chemistry, Institute of Medical Biology, Polish Academy of Sciences, Lodz, Poland; 2 Department of Pediatrics, Immunology, and Nephrology, Polish Mother's Memorial Hospital Research Institute, Lodz, Poland; 3 3rd Department of Pediatrics, Polish Mother’s Memorial Hospital Research Institute, Lodz, Poland; 4 Department of Perinatology and Gynecology, Polish Mother’s Memorial Hospital Research Institute, Lodz, Poland; 5 Scientific Laboratory of the Center of Medical Laboratory Diagnostics, Polish Mother's Memorial Hospital Research Institute, Lodz, Poland; 6 2nd Department of Obstetrics and Gynecology, Warsaw Medical University, Warsaw, Poland; University of San Francisco, UNITED STATES

## Abstract

Toll-like receptor 9 (*TLR9*) recognizes non-methylated viral CpG-containing DNA and serves as a pattern recognition receptor that signals the presence of human cytomegalovirus (HCMV). Here, we present the genotype distribution of single-nucleotide polymorphisms (SNPs) of the *TLR9* gene in infants and the relationship between *TLR9* polymorphisms and HCMV infection. Four polymorphisms (-1237T/C, rs5743836; -1486T/C, rs187084; 1174G/A, rs352139; and 2848C/T, rs352140) in the *TLR9* gene were genotyped in 72 infants with symptomatic HCMV infection and 70 healthy individuals. SNP genotyping was performed by using polymerase chain reaction-restriction fragment length polymorphism (PCR-RFLP). Digested fragments were separated and identified by capillary electrophoresis. The HCMV DNA copy number was measured by a quantitative real-time PCR assay. We found an increased frequency of heterozygous genotypes *TLR9* -1486T/C and 2848C/T in infants with HCMV infection compared with uninfected cases. Heterozygous variants of these two SNPs increased the risk of HCMV disease in children (*P* = 0.044 and *P* = 0.029, respectively). In infants with a mutation present in at least one allele of -1486T/C and 2848C/T SNPs, a trend towards increased risk of cytomegaly was confirmed after Bonferroni’s correction for multiple testing (*Pc* = 0.063). The rs352139 GG genotype showed a significantly reduced relative risk for HCMV infection (*Pc* = 0.006). In contrast, the -1237T/C SNP was not related to viral infection. We found no evidence for linkage disequilibrium with the four examined *TLR9* SNPs. The findings suggest that the *TLR9* -1486T/C and 2848C/T polymorphisms could be a genetic risk factor for the development of HCMV disease.

## Introduction

Human cytomegalovirus (HCMV), a member of the *Betaherpesvirinae* subfamily of herpesviruses, is a ubiquitous pathogen with a seroprevalence of 45–100% [1]. Primary HCMV infection is usually asymptomatic in healthy individuals and is followed by a persistent infection. In contrast, HCMV is a major reason of multiorgan disease in immunocompromised patients and a leading cause of congenital infection, occurring in 0.5–2% of pregnancies in the United States and Europe [2, 3]. Among congenitally infected neonates, approximately 10–15% exhibit signs and symptoms of disease at birth, and these symptomatic infants have an increased risk for sensorineural hearing loss and central nervous system damage [4, 5]. However, the factors that dictate whether HCMV infection is asymptomatic or symptomatic are not clear. Innate immunity plays a crucial role in preventing the acquisition of HCMV infections, whereas its failure may contribute to an increased risk of infection. It is suggested that variations in the genes that modulate innate immune responses, including *TLRs* genes, may result in distinct clinical presentations of infection.

Toll-like receptors (TLRs) play an important role in the innate immune response to pathogens and have been implicated in infectious and autoimmune processes. There is evidence that interactions between some TLRs with viruses influence both the immune response and outcome of HCMV infection. HCMV activates host cells *via* multiple TLRs, predominantly those that reside on cell surface or in endosomes. TLR2 is pattern recognition receptor (PRR) for HCMV glycoproteins B (gB) and gH [6]. TLR2 and CD14 participate in HCMV attachment, uptake, and subsequent signaling, leading to the expression of pro-inflammatory cytokine genes [6, 7]. TLR4 induces THP-1 cells signaling via the TLR4/MD2/CD14 complex which initiates and regulates additional downstream signaling [8]. It has been also demonstrated that TLR4-ligand complexes enhance the ability of dendritic cells to present viral antigen [9]. Endosomal TLR3, TLR7, and TLR9 are involved in HCMV-elicited signaling, which leads to a pro-inflammatory and antiviral response, such as type I interferons (IFNs) [6, 7, 10]. TLR3 recognizes dsRNA, also generated during the life cycle of viruses, whereas TLR7 and 8 recognize single-stranded RNA (ssRNA). TLR9, localized intracellularly within endolysosomes, recognizes unmethylated CpG dinucleotide motifs located in viral DNA and plays a central role in host defense against viral infection [11, 12]. HCMV strongly up-regulates *TLR9* mRNA levels in human fibroblasts [13]. TLR9 mediates the recognition of murine CMV (MCMV), as evidenced by experiments using a mutated form of TLR9. A missense mutation in the receptor domain of the *TLR9* gene (TLR9CpG1) can be induced by N-ethyl-N-nitrosourea and results in unresponsiveness to CpG-containing oligonucleotides. Mice homozygous for the TLR9CpG1 allele are highly susceptible to infection with MCMV and display impaired (infection-induced) IFN-α/ß secretion and NF-κB activation [14]. The TLR9-mediated activation of MyD88 and TLR3-dependent induction of TRIF signaling are activated *in vivo* upon inoculation of MCMV, leading to type I IFN production. Notably, neither of the pathways alone—in the absence of the other—offers complete protection against MCMV infection, but they instead act in an additive or codependent manner [14].

Little is known about the role of TLR polymorphisms in the pathogenesis of HCMV infection in humans. The R753Q single-nucleotide polymorphism (SNP) in the *TLR2* gene was shown to be associated with increased HCMV replication and disease in transplant recipients [15, 16]. *In vitro* experiments showed that the R753Q SNP abolishes TLR2-mediated immune signaling in response to HCMV [17]. No association between *TLR2* Arg753Gln SNP with HCMV infection was detected in both infants and immunocompromised adult patients [18]. Although the relationship between the mutation in the highly polymorphic *TLR4* gene and the incidence of HCMV disease has been described in transplant patients [19], no effect of the *TLR4* Asp299Gly SNP on viral infection in infancy was found [18]. Few studies have described the association between *TLR9* polymorphisms and HCMV infection [20–22]. Increasing studies have found that specific *TLR* SNPs have an association with congenital HCMV infection [23, 24]. Taniguchi et al. [23] found that the homozygous CC genotype of the 1350T/C SNP (rs3804100) in the *TLR2* gene was associated with congenital HCMV infection, while no associations between *TLR4* and *TLR9* SNPs with HCMV infection and disease in infants were found. Recently, *TLR4* and *TLR9* polymorphisms were found to play a role in the development of congenital HCMV infection in fetuses and neonates [24].

Our study explored the genotype distribution of *TLR9* -1237T/C (rs5743836), -1486T/C (rs187084), 1174G/A (rs352139), and 2848C/T (rs352140) SNPs in infants and the correlation between polymorphisms in the *TLR9* gene and HCMV infection in infants.

## Materials and Methods

### Ethics statement

The study protocols were approved by the Bioethics Committee of the Medical University of Lodz (RNN/120/09/KE), and the Ethics Committee of the Polish Mother’s Memorial Hospital Research Institute. Written informed consents were obtained from all parents or guardians on behalf of the children involved in the study.

### Study population

Between 2008 and 2011, 72 infants were selected as HCMV-positive at the 3rd Department of Pediatrics of the Polish Mother’s Memorial Hospital Research Institute in Lodz, Poland. HCMV infection was confirmed by HCMV DNA (*UL55* gene) detection in whole-blood and/or urine samples after 3 weeks of life and by the presence of HCMV-specific antibodies. Because infants were examined after that time, children were classified as having postnatal or unproven congenital HCMV infection. However, clinical data suggest that the majority of the studied infants were infected congenitally (Table 1). Serum samples from the infants were assessed for anti-HCMV IgG and IgM antibodies with the use of CLIA LIASON CMV IgM and IgG assays (DiaSorin, Sallugia, Italy). HCMV-positive infants were compared with 70 healthy newborn infants (seronegative and aviremic), which were used as controls for the association studies. All of the study subjects were Caucasians and there were no ethnic differences between the cases and control group.

**Table 1:** Demographic and clinical characteristics of study subjects with HCMV infection.

Characteristics	
**Total No.**	72
**Mean ± SD age (months)**	3.9 ± 2.4
**Gender number; n (%)**^**a**^	
Female	30 (41.7)
Male	42 (58.3)
**Symptoms; n (%)**^**a**^	
Hematological disorders	28 (38.9)
Pneumonia	19 (26.4)
Jaundice	16 (22.2)
Neurological dysfunction	16 (22.2)
CNS damage	15 (20.8)
Psychomotor retardation	14 (19.4)
Liver damage	11 (15.3)
Hearing loss	7 (9.7)
IUGR	5 (6.9)
Hepatosplenomegaly	5 (6.9)
Hepatitis	5 (6.9)
Heart disease	3 (4.2)
Ocular defects	2 (2.8)
**Anti-HCMV serologic status**	
IgG positive, IgM negative	40 (55.5)
IgG positive, IgM positive	29 (40.3)
IgG negative, IgM negative	3 (4.2)

^a^ Values are the number of infants (%).

All infants with HCMV infection were symptomatic due to the selection bias. Clinical samples were collected from children at diagnosis or after exacerbation of cytomegaly symptoms. The children were classified as having a symptomatic infection based on clinical or laboratory findings, including hematological disorders (anemia, thrombocytopenia), pneumonia, neurological dysfunction, central nervous system damage, psychomotor retardation, liver damage, intrauterine growth restriction, and other symptoms; the classification was made after other causes had been excluded. Of the 72 patients, 30 (41.7%) were female and 42 (58.3%) were male, and their mean age at evaluation was 3.9 ± 2.4 months (median: 3.3 months, range: 1–12 months). The demographic and clinical characteristics of infants with HCMV infection were summarized in Table 1. Laboratory personnel were blinded to the demographic characteristics and clinical findings of the study subjects.

### Genotyping of *TLR9* polymorphisms

**Fig 1:**
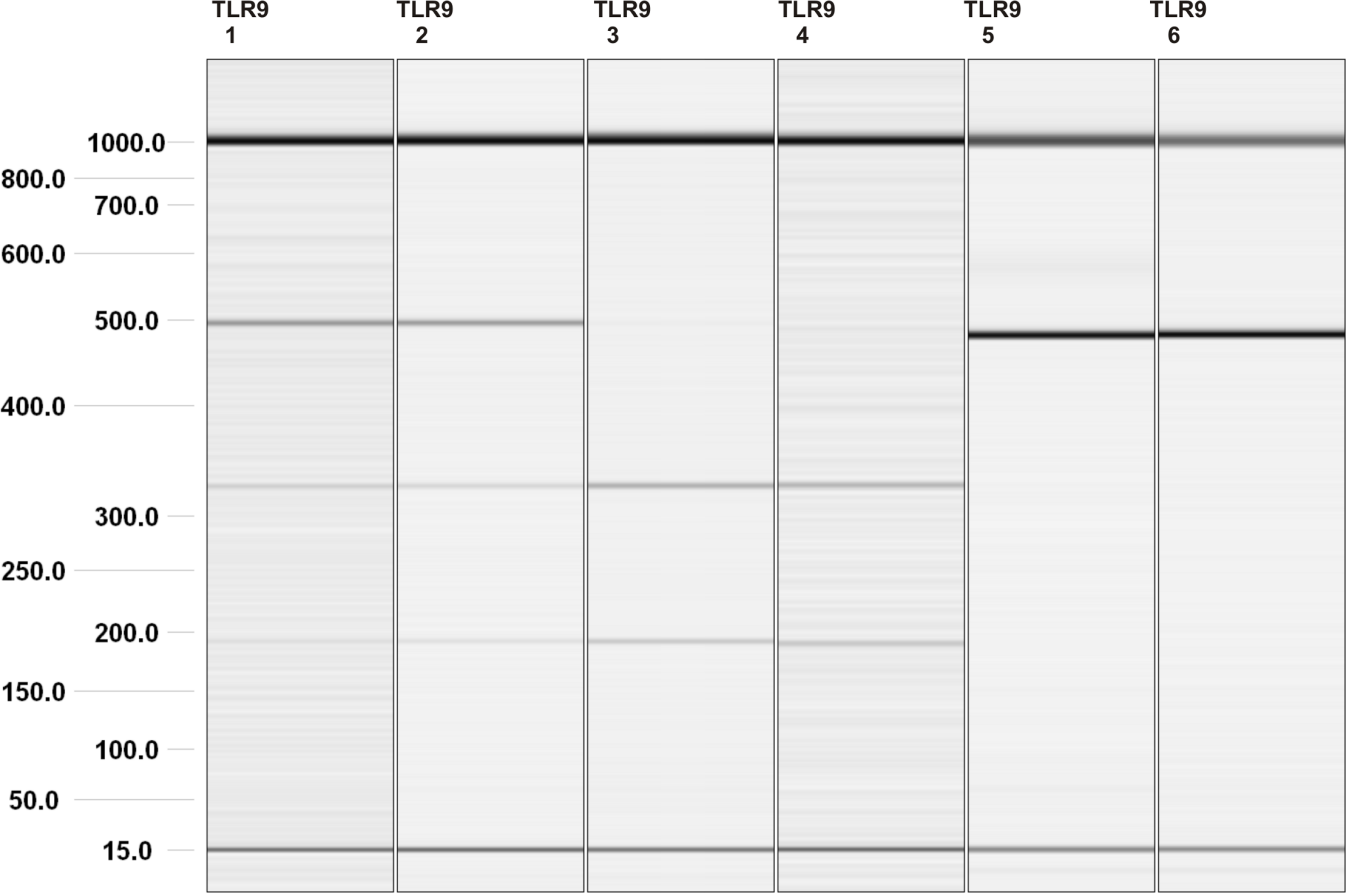
Visualization of selected PCR-RFLP products for *TLR9* -1486T/C genotyping. Gel image: 1 and 2, heterozygous TC genotype (192, 327, 490 bp); 3 and 4, TT genotype (192, 327 bp); 5 and 6, CC genotype (490 bp). Alignment markers (15 bp, 1 kbp).

All genotyping was performed on genomic DNA extracted from whole-blood samples using the QIAamp DNA Blood Mini Kit (Qiagen, Hilden, Germany) according to manufacturer’s recommendations. The concentration and purity of DNA were assessed using a NanoDrop 2000c UV-vis Spectrophotometer (Thermo Scientific, Wilmington, DE, USA). The identification of *TLR9* SNPs was performed by polymerase chain reaction-restriction fragment length polymorphism (PCR-RFLP) using primers described previously by Hamann et al. [25] (-1237T/C, rs5743836; -1486T/C, rs187084), Fang et al. [26] (1174G/A, rs352139) and Roszak et al. [27] (2848C/T, rs352140). Briefly, PCR was performed in a total volume of 50 μL containing 200 ng of genomic DNA, primers (1 μM each), dNTPs (2.5 mM), 10 x *Taq* buffer (100 mM Tris-HCl, 500 mM KCl, 0.8% Nonidet P40; pH 8.8), 2 mM MgCl_2_, and 1 U of *Taq* DNA Polymerase (Fermentas, Glen Burnie, MD, USA). The thermal cycling conditions for -1237T/C were 4 min at 95°C and 40 cycles each of 30 s at 95°C, 20 s at 50°C, and 30 s at 72°C. The PCR parameters for -1486T/C and 2848C/T were as follows: 4 min at 95°C and 35 cycles each of 30 s at 95°C, 20 s at 60°C, and 30 s at 72°C. The final extension in both PCR profiles was at 72°C for 5 min. The reactions were carried out using a Veriti thermal cycler (Applied Biosystems, Foster City, CA, USA). The PCR-amplified fragments corresponding to the *TLR9* -1237T/C, -1486T/C, 1174G/A, and 2848C/T polymorphisms were digested with the restriction enzymes MvaI, AflII, FspBI, and Bsh1236I, respectively (Fermentas, Hanover, MD, USA). The digested fragments were separated using the QIAxcel system (Qiagen). The *TLR9* SNPs variants were identified by different fragment lengths (Table 2; Fig 1). The results were confirmed by the direct sequencing of selected samples of each *TLR9* genotype using the MiSeq system (Illumina, San Diego, CA, USA). The results of the PCR-RFLP and sequencing methods were in agreement.

**Table 2:** Restriction enzymes and length of the restriction fragments.

SNP	Restriction enzyme	Genotype	Length of the restriction fragments, base pairs (bp)
**-1237T/C**	MvaI	TT	29 bp, 113 bp
		TC	29 bp, 113 bp, 142 bp
		CC	142 bp
**-1486T/C**	AflII	TT	192 bp, 327 bp
		TC	192 bp, 327 bp, 490 bp
		CC	490 bp
**1174G/A**	FspBI	GG	206 bp
		GA	28 bp, 178 bp, 206 bp
		AA	28 bp, 178 bp
**2848C/T**	Bsh1236I	CC	114 bp, 217 bp
		CT	114 bp, 217 bp, 331 bp
		TT	331 bp

### Assessment of HCMV replication

Real-time PCR quantification of the HCMV DNA copy number in DNA isolates from whole-blood and urine samples was performed by using a 7900HT Fast Real-Time PCR System (Applied Biosystems) [28]. For amplification of the HCMV genome, a pair of primers and a TaqMan probe labeled at the 5’ end with FAM (6-carboxyfluoroscein) and at the 3’ end with TAMRA (6-carboxytetramethylrhodamine) targeting the gB sequence were used. The primers and probe used were the following: primer gB1, 5’-GAGGACAACGAAATCCTGTTGGGCA-3’, primer gB2, 5’-GTCGACGGTGGAGATACTGCTGAGG-3’, and probe, 5’-CAATCATGCGTTTGAAGAGGTAGTCCA-3’. The samples for real-time PCR were prepared in a volume of 25 μL containing 5 μL of the DNA extract or calibrated plasmid dilution (standard curve), 0.4 μM of each primer, 0.2 μM of the fluorogenic probe, and the TaqMan Universal PCR Master Mix (Applied Biosystems). The PCR amplification reaction used the condition: 95°C for 10 min, 60 cycles at 95°C for 15 s, followed by 60°C for 1 min. Standard curves for the plasmid DNA were obtained from serial 10-fold dilutions from 10^5^ to 5. The number of target copies was determined from the threshold cycle CT.

### Statistical analysis

The calculation of Hardy-Weinberg equilibrium (HWE), linkage disequilibrium (LD) and additional genotype and haplotype associations with HCMV infection status were performed by using the SNPSTATS program (http://bioinfo.iconcologia.net/index.php?module=Snpstats) [29]. The association between the polymorphisms and HCMV infection was estimated using an odds ratio (OR) and 95% confidence intervals (95% CIs) with unadjusted and adjusted multivariate models. *P* values were corrected (*Pc*) for multiple testing with the Bonferroni’s correction and the *Pc*-values of ≤ 0.05 were considered to be significant. Logistic regression models to evaluate the association between the *TLR* genotype and the symptoms was performed using the SPSS statistical software package for Windows 17.0 (SPSS, Chicago, IL, USA). The distribution of genotypes and alleles in the patient groups was examined by using the Fisher’s exact test. The Mann-Whitney U test was used to study the association between *TLR* polymorphisms and viral load. The level of significance for all statistical tests was defined as *P* ≤ 0.05.

## Results

### Frequency of *TLR9* SNPs in infants

*TLR9* -1237T/C, -1486T/C, 1174G/A, and 2848C/T SNPs were typed in 142 infants, including the 72 subjects with HCMV infection. Almost all individuals (139/142, 97.9%) possessed the wild-type -1237TT genotype, while the CC genotype was only found in three HCMV-infected cases (3/72, 4.2%; *P* > 0.05). As the -1237T/C SNP is non-polymorphic in our population, it was excluded from further analyses. The distribution of the -1486T/C, 1174G/A, and 2848C/T SNPs was different between HCMV-infected and uninfected infants (see Table 3). In infants with HCMV infection, the frequencies of the TT, TC, and CC genotypes at the -1486T/C SNP were 22.2%, 62.5%, and 15.3%, respectively. The wild-type TT genotype was detected in 40.0% and the heterozygous TC genotype in 45.7%, while the homozygous CC genotype was detected in 14.3% of healthy individuals. Similar genotype frequencies in HCMV-infected and uninfected infants were observed in the *TLR9* 2848C/T SNP (Table 3). The frequency of wild-type genetic variants of both -1486T/C and 2848C/T SNPs was significantly higher in uninfected infants than in HCMV-infected cases (*P* = 0.029; Fisher’s exact test). It should be noted that, the heterozygous variant carriers of the 2848C/T and -1486T/C SNPs were detected more frequently among infants with HCMV infection (*P* = 0.044 and *P* = 0.064, respectively; Fisher’s exact test). In case of 1174G/A SNP, the frequencies of the GG, GA, and AA genotypes in children with HCMV infection were 1.4%, 40.3%, and 58.3%, respectively. The GG genotype at this SNP was identified more frequently in uninfected than HCMV-infected infants (14.3% vs 1.4%; *P* = 0.004; Fisher’s exact test). In the group of infants without HCMV infection, the frequencies of genotypes at all *TLR9* SNPs were in HWE (*P* > 0.05; Table 4).

**Table 3:** The distribution of genotypes frequencies of *TLR9* SNPs in infants and relationship between polymorphisms and the risk of HCMV infection.

*TLR9* SNPs	Model	Genotype	Genotype frequencies; n (%)^a^		Unadjusted			Adjusted^b^		
			HCMV- infected	Uninfected	OR (95% CI)	*P*-value	*Pc-*value^c^	OR (95% CI)	*P*-value	*Pc-*value^c^
**-1486T/C**	Codominant	TT	16 (22.2)	28 (40.0)	1.00	0.063	0.189	1.00	**0.012**	**0.036**
		TC	45 (62.5)	32 (45.7)	2.46 (1.15–5.28)			4.23 (1.53–11.67)		
		CC	11 (15.3)	10 (14.3)	1.92 (0.67–5.52)			2.80 (0.73–10.72)		
	Dominant	TT	16 (22.2)	28 (40.0)	1.00	**0.021**	0.063	1.00	**0.004**	**0.012**
		TC-CC	56 (77.8)	42 (60.0)	2.33 (1.12–4.86)			3.89 (1.45–10.46)		
	Recessive	TT-TC	61 (84.7)	60 (85.7)	1.00	0.870	ns	1.00	0.960	ns
		CC	11 (15.3)	10 (14.3)	1.08 (0.43–2.74)			1.03 (0.34–3.07)		
	Overdominant	TT-CC	27 (37.5)	38 (54.3)	1.00	**0.044**	0.132	1.00	**0.010**	**0.030**
		TC	45 (62.5)	32 (45.7)	1.98 (1.01–3.87)			2.87 (1.26–6.52)		
**1174G/A**	Codominant	AA	42 (58.3)	30 (42.9)	1.00	**0.005**	**0.015**	1.00	**0.005**	**0.015**
		GA	29 (40.3)	30 (42.9)	0.69 (0.35–1.38)			0.71 (0.32–1.58)		
		GG	1 (1.4)	10 (14.3)	0.07 (0.01–0.59)			0.00 (0.00-NA)		
	Dominant	AA	42 (58.3)	30 (42.9)	1.00	0.065	0.195	1.00	0.110	0.330
		AG-GG	30 (41.7)	40 (57.1)	0.54 (0.28–1.04)			0.53 (0.24–1.16)		
	Recessive	AA-AG	71 (98.6)	60 (85.7)	1.00	**0.002**	**0.006**	1.00	**0.002**	**0.06**
		GG	1 (1.4)	10 (14.3)	0.08 (0.01–0.68)			0.00 (0.00-NA)		
	Overdominant	AA-GG	43 (59.7)	40 (57.1)	1.00	0.760	ns	1.00	0.890	ns
		AG	29 (40.3)	30 (42.9)	0.90 (0.46–1.75)			0.94 (0.43–2.06)		
**2848C/T**	Codominant	CC	16 (22.2)	28 (40.0)	1.00	**0.049**	0.147	1.00	**0.014**	**0.042**
		CT	43 (59.7)	29 (41.4)	2.59 (1.20–5.63)			4.02 (1.43–11.28)		
		TT	13 (18.1)	13 (18.6)	1.75 (0.65–4.68)			3.59 (1.07–12.00)		
	Dominant	CC	16 (22.2)	28 (40.0)	1.00	**0.021**	0.063	1.00	**0.004**	**0.012**
		CT-TT	56 (77.8)	42 (60.0)	2.33 (1.12–4.86)			3. 89 (1.45–10.46)		
	Recessive	CC-CT	59 (81.9)	57 (81.4)	1.00	0.940	ns	1.00	0.470	ns
		TT	13 (18.1)	13 (18.6)	0.97 (0.41–2.26)			1.41 (0.56–3.60)		
	Overdominant	CC-TT	29 (40.3)	41 (58.6)	1.00	**0.029**	0.087	1.00	**0.046**	0.138
		CT	43 (59.7)	29 (41.4)	2.10 (1.07–4.09)			2.21 (1.01–4.85)		

^a^ Values are the number of examined infants (%)

^b^ Adjusted analysis was carried out for HCMV DNA copy number in whole-blood samples

^c^ The significance level after Bonferroni’s correction for multiple testing is 0.05

OR: odds ratio; 95% CI: 95% confidence interval; *P*, logistic regression model; *Pc*, value after Bonferroni’s correction accounting for multiple SNPs (raw *P*-value × 3); ns: not statistically significant; NA: not available.

**Table 4:** *TLR9* SNP variance from Hardy-Weinberg equilibrium.

*TLR9* SNPs	Infant group	Genotype/allele frequencies; n^a^					*P*-value
**-1237T/C**		TT	TC	CC	T	C	
	HCMV-infected	69	0	3	138	6	<0.0001
	Uninfected	70	0	0	140	0	1
**-1486T/C**		TT	TC	CC	T	C	
	HCMV-infected	16	45	11	77	67	0.056
	Uninfected	28	32	10	88	52	1
**1174G/A**		GG	GA	AA	G	A	
	HCMV-infected	1	29	42	31	113	0.16
	Uninfected	10	30	30	50	90	0.61
**2848C/T**		CC	CT	TT	C	T	
	HCMV-infected	16	43	13	75	69	0.16
	Uninfected	28	29	13	85	55	0.32

^a^ Values are the number of observed genotypes/alleles

*P*, chi-square test.

In the examined infants, the frequencies of the wild-type -1486 T, 1174 G and 2848 C alleles were 58.1%, 28.5% and 56.3%, respectively, while the frequencies of the -1486 C, 1174 A and 2848 T alleles were 41.9%, 71.5% and 43.7%, respectively. In both HCMV-infected and uninfected infant groups, no significant differences in the frequency of *TLR9* -1486T/C and 2848C/T alleles were observed (Table 5, *P* > 0.05). In contrast, the A allele of 1174G/A SNP was detected more frequently in infants with HCMV infection compared with uninfected cases (*P* = 0.009). Among HCMV-infected infants, in the -1237 locus, the C alleles were significantly more frequent than in uninfected individuals (*P* = 0.030).

**Table 5:** The distribution of allele frequencies of *TLR9* SNPs in infants with and without HCMV infection.

*TLR9* SNPs	Allele	Allele frequencies; n (%)^a^		*P*-value
		HCMV-infected	Uninfected	
**-1237T/C**	T	138 (95.8)	140 (100)	**0.030**
	C	6 (4.2)	0 (0)	
**-1486T/C**	T	77 (53.5)	88 (62.90)	0.119
	C	67 (46.5)	52 (37.1)	
**1174G/A**	G	31 (21.5)	50 (35.7)	**0.009**
	A	113 (78.5)	90 (64.3)	
**2848C/T**	C	75 (52.1)	85 (60.7)	0.153
	T	69 (47.9)	55 (39.3)	

^a^ Values are the number of alleles (%)

*P*, Fisher’s exact test.

### *TLR9* polymorphisms associated with HCMV infection

Statistically significant differences between infants with and without HCMV infection were observed for the *TLR9* -1486T/C, 1174G/A, and 2848C/T polymorphisms (see Table 3). For the -1486T/C, 1174G/A, and 2848C/T SNPs, the wild-type genotypes occurred more frequently in the uninfected infants compared to the HCMV-infected group. When considered separately, *TLR9* SNPs were significantly associated with risk of HCMV infection. Heterozygous and homozygous CC genotypes at the -1486 locus were associated with a 2-fold increased risk of HCMV infection (OR 2.33, 95% CI 1.12–4.86, *P* = 0.021 in dominant model). Similarly, at least a 2-fold increased risk of infection was found for the heterozygous and homozygous TT genotypes at the 2848 locus in almost all genetic models (OR 2.33, 95% CI 1.12–4.86, *P* = 0.021 and OR 2.10, 95% CI 1.07–4.09, *P* = 0.029 in the dominant and overdominant models, respectively). These two SNPs showed a trend to association with HCMV infection after Bonferroni’s correction for multiple testing (*Pc* = 0.063). In infants with double heterozygous -1486T/C and 2848C/T polymorphisms, a trend towards increased risk of cytomegaly was found (OR 1.95, 95% CI 0.92–4.13, *P* = 0.077). In contrast, the GG genotype of the 1174G/A SNP compared to GA and AA genotypes decreased the risk of HCMV infection (OR 0.08, 95% CI 0.01–0.68, *P* = 0.002 in recessive model). These *P* value remained significant even after Bonferroni's correction (*Pc* = 0.006).

For the *TLR9* -1486T/C and 2848C/T SNPs, a mutation present in at least one allele may lead to HCMV infection in infants. No association between specific *TLR9* genotypes and the presence of specific symptoms in HCMV-infected children was found.

### Associations between *TLR9* SNPs and HCMV load

**Fig 2:**
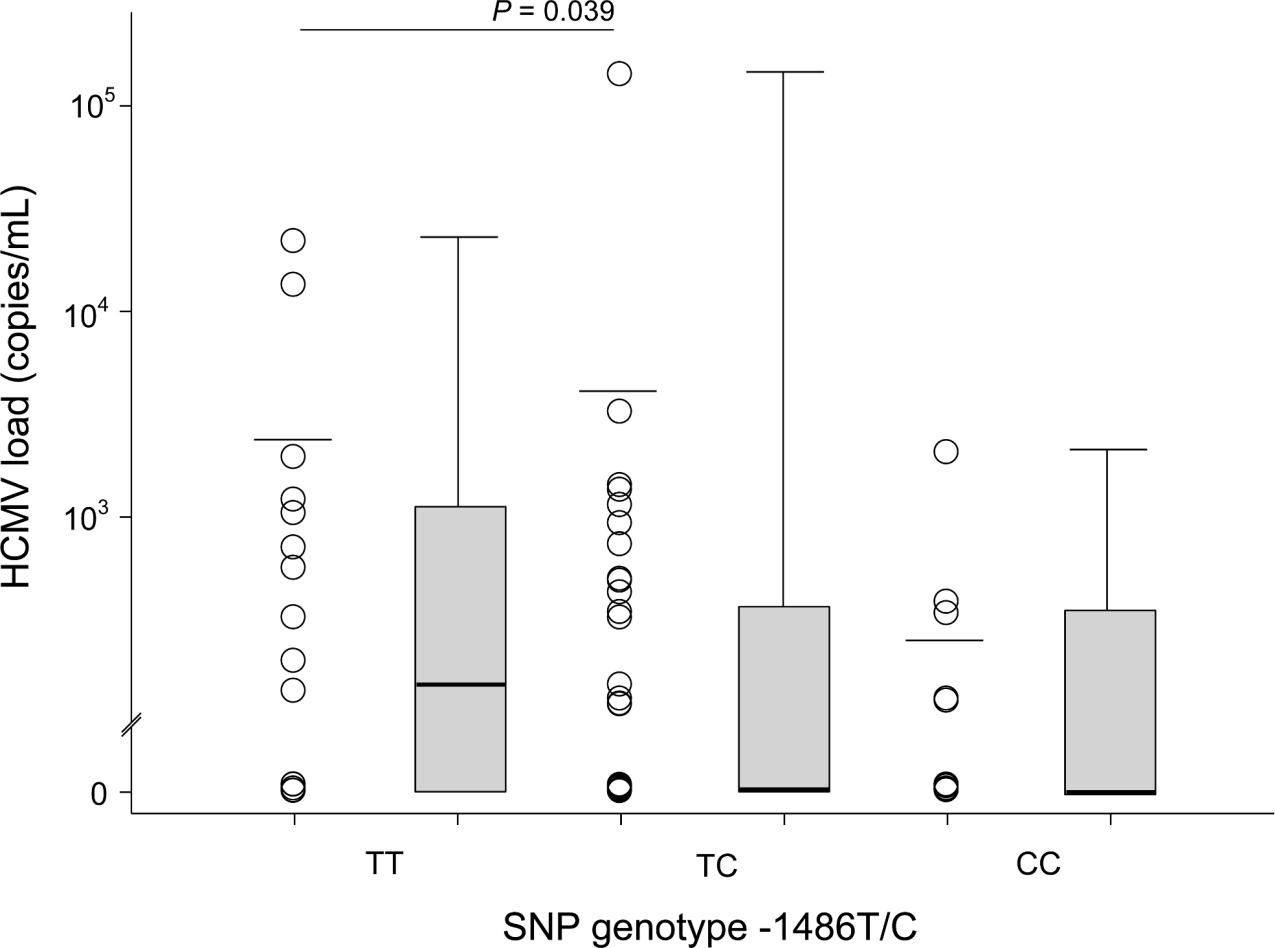
Comparison of viremia levels in HCMV-infected infants without or with the *TLR9* -1486T/C SNP (N = 72). Bars in the scatter dot plot represent the mean viral loads. Bars in the box plots represent median viral loads, upper and lower borders represent the 25th and 75th percentiles, and whiskers represent the minimum to maximum values. *P* ≤ 0.05, Mann-Whitney U test.

We correlated the SNPs with HCMV DNA concentration in peripheral blood during exacerbation of cytomegaly symptoms. The viremia levels ranged from 0 to 1.41 × 10^5^ copies/mL (mean 2.73 × 10^3^ ± 1.68 × 10^4^ copies/mL) in HCMV-infected infants. However, the mean copy number of HCMV DNA was statistically higher in blood samples from infants with the heterozygous variant of *TLR9* -1486T/C than those with the wild-type genotype (Fig 2, *P* = 0.039). In addition, the children with heterozygous and homozygous recessive genotypes at the -1486 and 2848 locus had almost 4-fold increased risk of HCMV disease in an adjusted model that included the HCMV DNA copy number (OR 3.89, 95% CI 1.45–10.46, *P* = 0.004 in dominant model; Table 3). This association reach statistical significance after Bonferroni’s correction (*Pc* = 0.012). No association was observed between the HCMV DNAemia and *TLR9* 1174G/A SNP (*P* > 0.05).

### Haplotype analysis

Multiple-SNP analysis showed that the most common haplotype for *TLR9* -1237T/C, -1486T/C, 1174G/A and 2848C/T was TCAC (21.62% and 18.46% for HCMV-infected and uninfected cases, respectively). The CCAT, CTGC, and CCAC haplotypes were detected only in infants with HCMV infection, while CTAC haplotype was not detected in any children. We found an association between -1486T/C and 1174G/A SNPs (*Pc* < 0.001). Linkage disequilibrium analysis revealed that -1237T/C, -1486T/C, 1174G/A, and 2848C/T SNPs were not in LD with each other (correlation coefficient r^2^ < 0.2). In addition, the haplotypes were not observed to influence the risk for HCMV infection.

An additional multiple-SNP analysis for *TLR9* -1237T/C, -1486T/C, and 2848C/T showed that the TCT haplotype was observed at a lower frequency in both infant groups (24.3% and 14.7% for HCMV-infected and uninfected cases, respectively) and it was associated with 2.5-fold increased the risk of HCMV infection (OR 2.47, 95% CI 1.14–5.33, *P* = 0.023). However, association did not reach statistical significance after Bonferroni’s correction (*Pc* > 0.05).

## Discussion

In this study, we demonstrated that the heterozygous genotype of *TLR9* -1486T/C and 2848C/T occurred more frequently in infants with HCMV infection than in uninfected cases. The heterozygous or homozygous recessive genotypes of the *TLR9* SNPs were consistently associated with a 2-fold higher risk of HCMV infection and disease development. This study provides the first demonstration that the 2848C/T and -1486T/C genotype might be important markers of HCMV infection. Furthermore, the GG genotype of the 1174G/A locus showed a significantly reduced risk for HCMV infection. No association between a polymorphism in the promoter of the *TLR9* gene (-1237T/C) and HCMV infection was found. We speculate that genetic variations in *TLR9* lead to a functional deficiency of the receptor signaling pathway and they are partially responsible for the development of cytomegaly.

TLR9 has been implicated in the recognition of different families of DNA viruses, all of which contain genomes rich in CpG DNA motifs, including herpesviruses [30–33]. The intracellular localization of TLR9 is critical for the discrimination of self and non-self nucleic acids. TLR9 recognizes unmethylated CpG dinucleotide motifs in DNA, a feature that is enriched in viruses and not present in host genomes. After stimulation with CpG DNA, TLR9 redistributes from the endoplasmic reticulum to lysosomes, where the receptor undergoes proteolytic cleavage of its ectodomain. Only the cleaved receptor is able to recruit MyD88 [34, 35], which signals through a protein complex consisting of TRAF6 and IL-1 receptor-associated kinase 1/4 (IRAK1/4), leading to the activation of IRF7, NF-ĸB, and mitogen-activated protein kinase [36–38]. Thus, TLR9 activates the production of type I IFNs and inflammatory cytokines. Because TLR9 signaling leads to the activation of IRF7, the recognition of herpesviruses by dendritic cells leads to the expression of type I IFNs [30, 32, 39].

We studied the -1237T/C and -1486T/C SNPs, which are located within the putative promoter region, 1174G/A SNP located in intron 1, and the 2848C/T SNP located in exon 2 of *TLR9* gene. *TLR9* polymorphisms that are located in the promoter region most likely alter the functional ability of the receptor, as reported in other studies [40–42]. These *TLR9* SNPs have been reported to show high heterozygosity in other population [43]. We observed a low prevalence of the -1237T/C SNP in the examined population and an incidence of HCMV disease in individuals carrying the *TLR9*–1237 C allele. An analysis of the second polymorphism of the *TLR9* promoter -1486T/C revealed that the mutation present in at least one allele enhanced risk of HCMV disease. There are no functional data available for the *TLR9* -1486T/C polymorphism but it likely alters the function of the promoter. The combination of the C allele at position -1486 with a G allele at position 1174 has the ability to downregulate *TLR9* expression, and the C allele predisposes patients to systemic lupus erythematosus [44]. Kikuchi et al. [45] reported that the 2848G/A SNP was associated with alterations in gene expression. This polymorphism does neither results in an amino acid change nor modification of a regulatory site, implying functional linkage with another proximal SNPs [46]. We found that infants with double heterozygous 2848C/T and -1486T/C genotypes had a trend towards increased risk of HCMV infection. Similar to the -1486T/C SNP, a mutation present in at least one allele of 2848C/T was associated with HCMV infection. We speculate that genetic variations of *TLR9* that down regulate its expression could reduce the function of the innate immune response against HCMV infection. The previous study showed that the *TLR9* 2848 heterozygous status predisposes fetuses and newborns to HCMV infection and increases the risk of cytomegaly development [24]. Although the frequency of the heterozygous genotype of *TLR9* SNP rs352140 was also higher in Japanese children congenitally infected with HCMV, no significant association between genotype variants and viral infection was found [23]. The present study revealed a significant association between 2848C/T genotype and the higher risk of HCMV infection in infants with postnatal or unproven congenital HCMV infection. Our findings indicate no significant association of *TLR9* 1174G/A polymorphism with cytomegaly among Polish infants. Nevertheless, we observed that the 1174 GG carriers had a reduced risk of HCMV infection. The genotype and allele distribution of *TLR9* SNPs was comparable to that in other European populations [20, 27, 47–49] but was different than in Asian and American ethnic groups [48, 50–52]. These results indicate the existence of a geographic difference in *TLR9* genotypes that may explain the variability to pathogen susceptibility or other conditions under which TLR9 pathways may be involved.

Specific haplotypes for the *TLR9* gene might affect host defense mechanisms and influence the susceptibility or resistance to infections. *TLR9*–1486 C allele carriers are associated with an increased risk and poor prognosis of gastric carcinoma in the Chinese population [53], and the 2848C/T polymorphism may be associated with Hodgkin’s lymphoma [54] and cervical cancer [27, 55]. Previous studies have shown that *TLR9* polymorphisms are associated with various viral infections, including HIV-1 [56–60], HPV16 [55], and HCMV [23, 24]. Two *TLR9* SNPs, 1174G/A (rs352139) and 2848C/T, were linked to viral load and disease progression in HIV-1-infected adults [56, 57, 59]. A significant correlation between these genetic variants and the risk of mother-to-child transmission of HIV-1 infection was found [58]. Moreover, the heterozygous 2848 AG genotype and G haplotype were strongly associated with rapid disease progression in HIV-1-infected children [60]. The role of *TLR9* polymorphisms in HCMV infection is only beginning to be appreciated. It was previously reported that the -1237T/C mutated C allele was highly predictive of susceptibility to HCMV infection in stem cell transplant recipients [20]. However, there was no effect of *TLR9* -1237T/C and 2848C/T polymorphisms on HCMV infection in renal transplant recipients [22]. Two other SNPs in the *TLR9* gene in the donor, 1174G/A and 1635C/T (rs352140), influenced the risk of both acute graft-versus-host disease and HCMV reactivation in allogeneic hematopoietic recipients [21]. In contrast, no correlation was found between these SNPs and congenital HCMV infection or disease [23]. The current and previously described results indicated that *TLR9* polymorphisms have an impact on the development of HCMV disease.

Our finding that *TLR9* -1486T/C and 2848C/T SNPs are associated with HCMV infection suggests an important role of TLRs and TLR-mediated signaling in the pathogenesis and outcomes of cytomegaly. Studying SNPs among the receptors involved in viral recognition will be essential to define the genetic background associated with risk of HCMV infection and disease. Further studies that focus on gene expression and functional consequences of polymorphisms in cytomegaly are needed to determine the exact consequence of *TLR9* SNPs. Increased understanding of how *TLR9* polymorphisms affect congenital cytomegaly may provide a means of identifying high risk groups among newborns and their mothers.

In conclusion, it was found that the heterozygous genotypes of *TLR9* -1486T/C and 2848C/T SNPs as well as the homozygous AA genotype of the intronic 1174G/A SNP were prevalent in infants with HCMV infection. An association between the heterozygous and homozygous recessive genotypes of the -1486T/C and 2848C/T SNPs and predisposition to viral infection was found. Our observations provide new insight into the role of *TLR9* polymorphisms in HCMV infection and may suggest that the presence of the mutation in at least one allele may lead to disease development.
